# Visualizing the superfamily of metallo-β-lactamases through sequence similarity network neighborhood connectivity analysis

**DOI:** 10.1016/j.heliyon.2020.e05867

**Published:** 2021-01-02

**Authors:** Javier M. González

**Affiliations:** Instituto de Bionanotecnología del NOA (INBIONATEC), Consejo Nacional de Investigaciones Científicas y Técnicas, Universidad Nacional de Santiago del Estero (CONICET-UNSE), G4206XCP Santiago del Estero, Argentina

**Keywords:** Metallo-lactamase, Protein superfamily, Tanglegram, Sequence similarity network, Neighborhood connectivity

## Abstract

Protein sequence similarity networks (SSNs) constitute a convenient approach to analyze large polypeptide sequence datasets, and have been successfully applied to study a number of protein families over the past decade. SSN analysis is herein combined with traditional cladistic and phenetic phylogenetic analysis (respectively based on multiple sequence alignments and all-against-all three-dimensional protein structure comparisons) in order to assist the ancestral reconstruction and integrative revision of the superfamily of metallo-β-lactamases (MBLs). It is shown that only 198 out of 15,292 representative nodes contain at least one experimentally obtained protein structure in the Protein Data Bank or a manually annotated SwissProt entry, that is to say, only 1.3 % of the superfamily has been functionally and/or structurally characterized. Besides, neighborhood connectivity coloring, which measures local network interconnectivity, is introduced for detection of protein families within SSN clusters. This approach provides a clear picture of how many families remain unexplored in the superfamily, while most MBL research is heavily biased towards a few families. Further research is suggested in order to determine the SSN topological properties, which will be instrumental for the improvement of automated sequence annotation methods.

## 1 Introduction

The metallo-β-lactamase (MBL) superfamily comprises an ancient group of proteins found in all domains of life, sharing a distinctive αββα fold with a histidine-rich motif for binding of transition metal ions. Such characteristic αββα domain uniquely places the metal binding site at the bottom of a wide groove that evolved to accommodate varied substrates. The name was coined after the first superfamily members to be characterized: a group of zinc-dependent hydrolases produced by bacteria resistant to β-lactam antibiotics. These zinc-β-lactamases (ZBLs) hydrolyze the amide bond present in all β-lactams and thus render them ineffective. The first X-ray crystallographic report of a ZBL was that of BcII from *Bacillus cereus* 569/H/9 [1]. Despite its low resolution, the atomic model disclosed the new αββα fold and a single Zn(II) ion bound to a three-histidine motif, resembling the active site of carbonic anhydrases. Thus, BcII and ZBLs in general were believed to use a single Zn(II) ion to activate a water molecule for hydrolysis, paralleling the mechanism by which carbonic anhydrases catalyze carbon dioxide hydration. This hypothesis was soon questioned when the structure of ZBL CcrA from *Bacteroides fragilis* was published, disclosing a bimetallic zinc center, with the second zinc being coordinated to nearby Asp, Cys and His residues [2]. Besides, the second zinc was later found in *B. cereus* ZBL too [3, 4, 5], starting a decade-long controversy regarding the role of each zinc ion. Later on, it was found that monometallic ZBLs are rather exceptional and the hydrolysis reaction generally requires two Zn(II) ions [6, 7].

**Figure 1:**
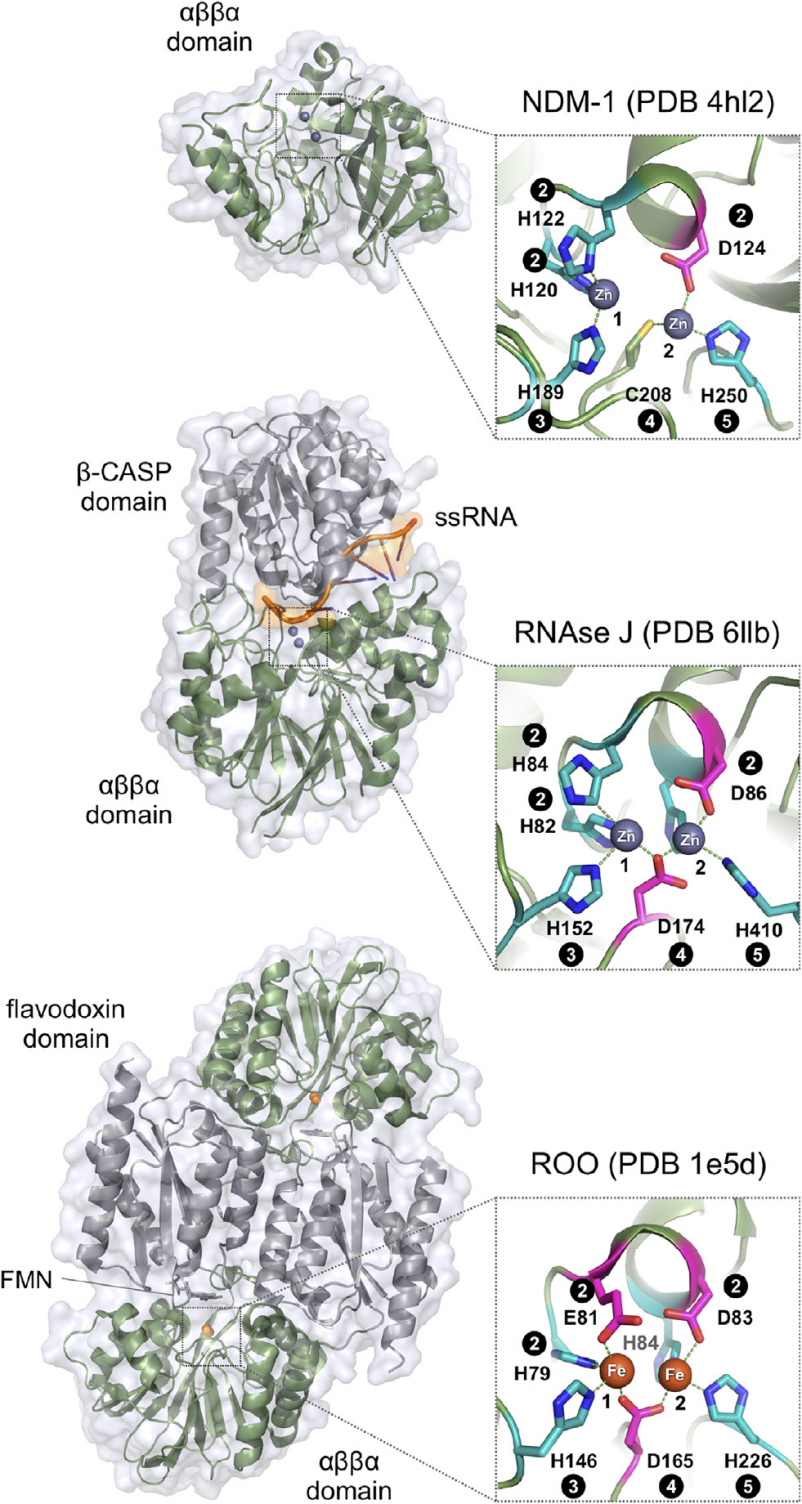
Structural diversity of MBLs. ZBLs like plasmid-borne *Klebsiella pneumoniae* NDM-1 (PDB 4hl2, *top*) comprise a single αββα domain (*green*), with the Zn(II) binding site at the bottom of an open groove, accessible to varied β-lactam antibiotics. B1 ZBLs exhibit unusual zinc ligands, including a cysteine residue, which is uncommon in catalytic Zn(II)-binding sites. Instead, habitual MBL metal ligand sets include only histidine and aspartic acid residues. For instance, RNAse J from *Methanolobus psychrophilus* (PDB 6llb, *middle*) comprises a phosphoesterase αββα domain and a β-CASP domain (*gray*) for single-stranded RNA binding (*orange*). Finally, MBL oxidoreductases such as the flavo-diiron protein ROO from *Desulfovibrio gigas* (PDB 1e5d, *bottom*) utilize non-heme Fe(II)/Fe(III) for catalysis, exhibiting a more acidic metal ligand set, in combination with an FMN-binding flavodoxin domain (*gray*), displaying a homodimeric quaternary structure. Metal ions are indicated as numbered spheres. Amino acid side chains follow the coloring scheme of Figure 2. Circled numbers indicate the corresponding motifs, as defined in Figure 2.

A great diversity of proteins evolved in the MBL superfamily by combining catalytic MBL domains and substrate recognition domains in a modular fashion. Subtle changes in the metal coordinating residue networks expand this diversity by enabling the coordination of different transition metals, particularly Zn(II), Mn(II), and Fe(II)/Fe(III) (Figure 1). Early attempts to build a systematic classification of the MBL superfamily were conducted by L. Aravind [8], as some of the very first applications of the PSI-Blast algorithm [9], who showed that many proteins other than ZBLs comprise the characteristic fold and histidine-rich metal-binding motif of MBLs, mapping key residues onto the structure of *B. cereus* ZBL. These observations were updated in 2001 by Daiyasu *et al.*, when additional crystal structures of MBL superfamily members were available [10]. At present, more than a hundred proteins have been shown to contain αββα domains through X-ray crystallography, whereas the InterPro 77.0 [11] database entry IPR001279 for the MBL superfamily includes about half a million members. Indeed, the MBL superfamily has grown astoundingly over the past 30 years, and an integrative revision is long overdue.

In recent years, protein families available in public databases have grown in number and size at unprecedented rates. Thus, improved methods for accurate analysis of large protein sequence datasets are urgently needed, since such a task is unattainable with the classical approach of multiple sequence alignment (MSA) plus phylogenetic tree calculation. A convenient approach introduced relatively recently by professor Babbitt group at UCSF is the construction of sequence similarity networks (SSN) [12]. SSNs comprise nodes representing a given set of polypeptide sequences interconnected with edges for a specified similarity cutoff value, and have been successfully applied to characterize a number of protein superfamilies in the past decade [13, 14, 15, 16, 17, 18, 19]. Nonetheless, identifying protein families within network clusters with missing experimentally-obtained functional or structural information is still an unsolved problem. Besides, the topological properties of SSN are largely unknown in comparison with classic models like random, small-world, and scale-free networks [20]. In this work, a large-scale MSA-based cladogram and a structure-based phenogram are calculated for the superfamily of metallo-β-lactamases in order to assist its phylogenetic reconstruction, providing a framework for an updated integrative revision. In addition, the neighborhood connectivity (NC) analysis [21] is introduced as an intuitive guide to search for uncharacterized new families within SSN clusters.

## 2 Materials and methods

### 2.1 Structural data harvesting and tanglegram calculation

All MBL protein sequences with available experimentally determined three-dimensional structure were retrieved from the Protein Data Bank (PDB) with the Dali Lite server [22], using structures PDB 2gmn and PDB 3i13 as queries. A set of 105 high-resolution structures was obtained after applying a 90 % sequence similarity cutoff. As well, an unrooted structural dendrogram was obtained for this set with the Dali Lite server all-against-all comparison tool, which calculates a distance matrix of *Z*-scores by aligning the structures all-against-all and outputs a dendrogram derived with the average linkage clustering method [23]. Next, the full amino acid sequence corresponding to each of these 105 structures were retrieved from the UniProt database [24], in order to avoid sequence artifacts like mutations and missing residues often found in PDB files. A structure-guided multiple sequence alignment (MSA) was calculated with Promals3D [25]. This MSA was manually edited with Jalview 2.9 [26] to discard highly gapped regions, by applying a 50 % alignment quality cutoff. The resulting MSA, comprising 105 sequences and 204 columns, was used to calculate a maximum likelihood cladogram with RAxML [27], running at the Cipres server [28]. A best-scoring bootstrapped tree was obtained after 1002 replicates, using the WAG substitution matrix as evolutionary model [29], and was displayed as a consensus cladogram by applying the 50 % majority rule. Finally, in order to compare the consensus sequence-based cladogram with the distance-based dendrogram topologies, a tanglegram matching corresponding taxa was calculated with the Neighbor Net Tanglegram algorithm [30], available in Dendroscope 3.5.9 [31], using the clade of B1&B2 zinc-β-lactamases as outgroup to root each tree. The tanglegram was adapted for display with FigTree 1.4.3 (available at http://tree.bio.ed.ac.uk/software/figtree/) and Corel Draw X7 (Corel). Protein structures were analyzed and graphically represented with PyMOL 1.8 (Schrödinger LLC).

### 2.2 Sequence data harvesting and SSN calculation

In order to prepare a representative sequence data sample of the MBL superfamily, the PF00753 Pfam database entry was selected as a starting point, which presently comprises 70,367 sequences (release Pfam 32.0, September 2018) [24]. The RP55 representative proteome MSA (62,213 sequences by 1,251 columns) was downloaded and manually edited with Jalview 2.9 [26], by removing truncated and misaligned sequences, highly gapped columns (more than 50 %); and deleting those sequences missing conserved positions corresponding to aspartic acid residues 29, 58, and 134 of human glyoxalase II, which was taken as a reference. The resulting MSA consisted of 55,076 sequences and 143 columns. Next, the full sequences present in this MSA set were retrieved from the UniProt 2019-10 database and reduced to a final set of 32,418 sequences, by applying a 70 % similarity cutoff with CD-Hit [32] and ensuring that all 105 sequences present in the tanglegram were included. A sequence similarity network (SSN) [12] was then calculated with this 32,418-sequence dataset, using the EFI-EST online tool [33]. The obtained representative node network comprised 15,292 nodes at 40 % sequence similarity, and 762,784 edges at 10–20 Blast pairwise similarity threshold. Topology network analysis was performed with NetworkAnalyzer 2.7 [34], as implemented in Cytoscape 3.7.1 [35]. Network statistics plots were prepared with SigmaPlot 12 (Systat Software). All figures were prepared with Corel Draw X7 (Corel).

## 3 Results and discussion

### 3.1 Unearthing ancestral relationships within the MBL superfamily

Tracing the evolutionary history of ancient protein superfamilies is often obscured by the inherent variability of amino acid sequences over long periods. Despite the divergence of primary structure, the three-dimensional fold of polypeptides is less sensitive to mutational events, retaining evolutionary information encoded in the arrangement of secondary structure elements. Thus, experimentally determined structures of proteins offer the possibility of common ancestry inference based on structural homology. Such *phenetic* methods are convenient for comparing proteins with similar folds but highly divergent amino acid sequences, in contrast to MSA-based *cladistic* methods, which are well suited to determine phylogenetic relationships between homologous proteins.

**Figure 2:**
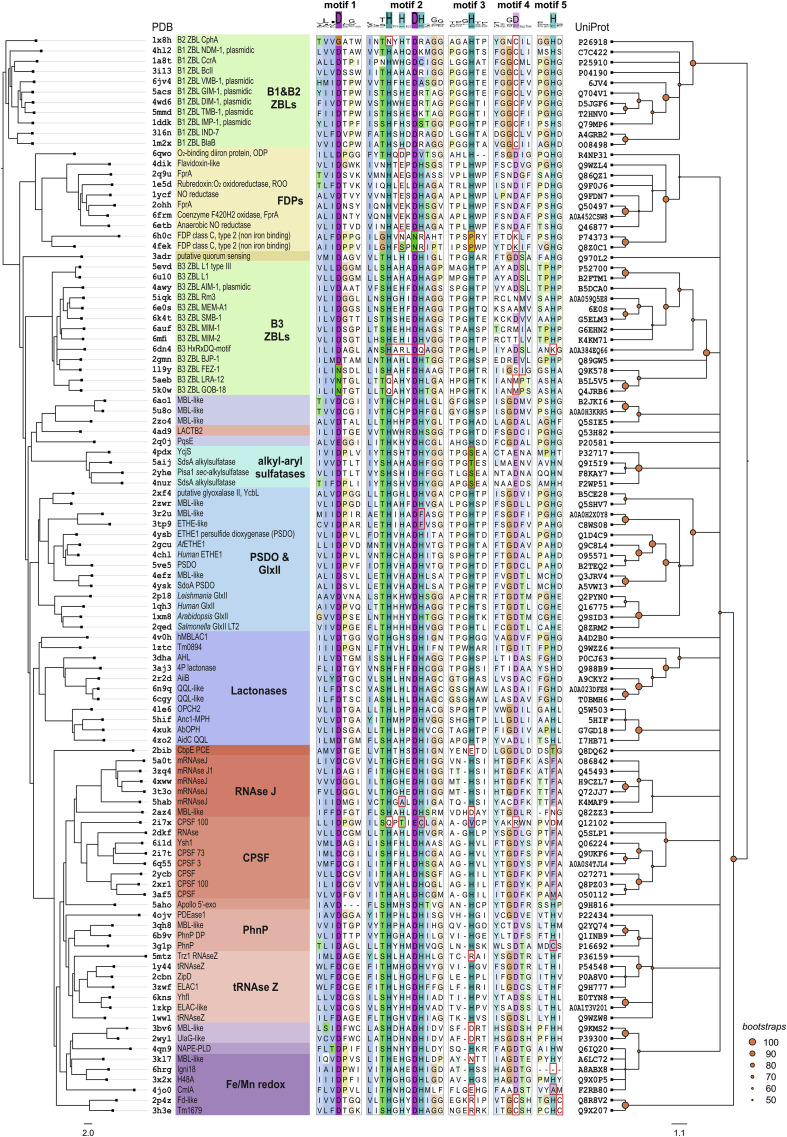
Structure-function tanglegram of the MBL superfamily. Structure-guided phenogram (*left*) and the maximum-likelihood MSA-based bootstrapped consensus cladogram (*right*) of 105 selected MBLs available in the Protein Data Bank. Note that the MSA includes only conserved amino acid residues in the αββα fold, *i.e.* it does not take into account additional domains. For each dendrogram, taxa are indicated as representative PDB entries (used for structural phenogram calculation) or UniProt entries (used for MSA and cladogram calculation), respectively. A short version of the MSA is provided, comprising the corresponding sequences sorted with the tanglegram, showing the five MBL fold conserved sequence motifs as histogram logos (*top*), along with short descriptions of common protein names and families (*colored boxes*). While motif 1 contains a conserved aspartic acid residue involved in stabilization of the MBL fold near the active site; motifs 2, 3, 4 and 5 usually contain metal-coordinating residues. In general, Fe(II)/Fe(III) binding sites typical of MBL oxidoreductases exhibit more acidic residues than Zn(II) binding sites, often found in MBL hydrolases. Distinctive residues of each protein family or group are indicated in the MSA as *red* boxes. Amino acid sequence lengths are variable between these motifs, ranging 6–503 residues before motif 1 (N-terminus); 9–77 residues between motifs 1 and 2; 3–23 residues between motifs 2 and 3; 11–65 residues between motifs 3 and 4; 14–241 residues between motifs 4 and 5; and 0–58 residues after motif 5 (C-terminus). Orange dots in consensus cladogram nodes indicate bootstrap branch support values higher than 50 %.

A structure-based approach for functional classification of MBLs was applied by Garau *et al.* in 2005, who used normalized root mean-square values as structural diversity estimates in order to calculate structure-guided phylogenies [36]. They conclude that structural similarity, as defined by differences in positions of Cα atoms of fitted homologous structures, is an acceptable estimate of evolutionary relatedness of proteins sharing comparable folds. A variant of this approach is herein employed, using the Dali *Z*-score as a more accurate estimate of structural similarity for a set of currently available experimental MBL structures. A distance matrix of Dali *Z*-scores comparing all-against-all full-length 105 selected MBL structures was used to construct the corresponding structural phenogram, that is, an unrooted tree whose branch lengths reflect structural similarity relationships between proteins, independently of their amino acid sequence. Next, the amino acid sequences of those 105 polypeptides were retrieved and aligned to construct a maximum-likelihood MSA-based bootstrapped unrooted consensus cladogram, whose topology reflects the sequence homology relationships between extant taxa according to a specific evolutionary model. Both dendrograms were then rooted using the B1&B2 ZBL clade as outgroup, since these enzymes are uniquely divergent MBLs due to their fast-evolving nature. The most distinctive feature of this outgroup is the presence of a Zn(II)-binding cysteine residue which is uncommon in catalytic Zn(II) sites, and has been shown to enable Zn(II) binding at limiting metal concentrations [7]. A tanglegram was then calculated with both trees, which consists of a graph of opposing dendrograms with lines connecting equivalent or corresponding taxa, rearranged so that the number of crossing connecting lines is minimal. This type of graph is widely used in Biology to illustrate processes like host-parasite, mutualistic, and symbiotic relationships, where both trees tend to comprise mirror images of each other, as a reflection of their shared topology and evolutionary history. Tanglegrams are used here to explore reciprocal similarities between structure and function of proteins. Since conserved structural features are substantiated by sequence adaptations to perform a specific function, sequence and structure can be assumed to evolve together, and should therefore give rise to dendrograms with the same topology. Crossing connectors between proteins would suggest that conserved residues typical of one group of proteins are found in a scaffold characteristic of different ones. Since the MSA consensus cladogram is not resolved at early nodes, a typical feature of phylogenies of divergent protein families, both trees can be rearranged so that no crossing connecting lines are needed between taxa (Figure 2).

### 3.2 Phenetic and cladistic considerations shed light on mutual MBL ancestors

ZBLs comprise a divergent polyphyletic group of MBLs, including subclasses B1, B2, and B3 [37]. It is important to note that, while ZBLs hydrolyze antibiotics by means of a metal-activated water molecule, most β-lactamases use a conserved serine residue in a completely different protein scaffold. In other words, the majority of β-lactamases are not metallic, and referring to ZBLs and MBLs in general simply as “β-lactamases” should be avoided, particularly when annotating these proteins in public databases. Besides, even though most members of the superfamily are devoid of β-lactamase activity, the acronym MBL has been adopted to annotate most members of the superfamily. The same convention is followed here to define any protein with at least one characteristic MBL domain, leaving the acronym ZBL to describe metallo-β-lactamases themselves.

As shown in the tanglegram and suggested previously [36, 38], B3 ZBLs form a phylogenetically distinct group as compared with B1&B2 enzymes, a clear example of how ZBL activity evolved twice within the superfamily. Motif 2 of B1, B2, and B3 ZBLs are characteristically of the form HxHxDX (where X is not a zinc ligand, typically Arg, Lys or small side chain residues), NxHxDR and HxHxDH, respectively. While B2 ZBLs are typically strict carbapenemases, B1 and B3 ZBLs display low substrate selectivity, and are able to hydrolyze all penicillins, cephalosporins and carbapenems of clinical use. Only monobactams remain insensitive to hydrolysis by ZBLs. Subclass B1 plasmid-borne ZBLs like IMP-1 (see Figure 2 for UniProt identifiers) became known in the ‘90s for their ability to hydrolyze carbapenems, the latest generation of β-lactam antibiotics available. 30 years later, pathogens expressing B1 enzymes like NDM-1 (Figure 1) still comprise one of the most cumbersome public health issues. In agreement with previous observations, B1&B2 enzymes are closely related and share a recent ancestor, along with a distinctive Zn(II)-binding cysteine at motif 4, supporting antibiotic resistance at limiting Zn(II) concentrations [7]. In contrast, B3 enzymes are typically chromosomal and replace this cysteine with residues unable to coordinate Zn(II) ions, like Ser, Ile, Val, Leu, and Met. In addition, all motif 2 histidines of B3 enzymes become zinc ligands, which is the usual scenario throughout the superfamily. A standard numbering scheme has been proposed for ZBLs [39], where metal-binding residues in motifs 2 to 5 are respectively: His/Gln116, His118, and His196 for Zn1; and Asp120, Cys221/His121, and His263 for Zn2 (*cf*. Figure 2). It is worth emphasizing that the HxHxDH motif is the hallmark of the superfamily, and such sequence diversity at motif 2 of ZBLs is rather unusual for a group of enzymes catalyzing the same reaction. This variability likely results from the strong selective pressure exerted by the comparably diverse set of β-lactam antibiotics currently in use.

Recently, new classification schemes have been proposed for ZBLs based on large-scale genomic and metagenomic data searches, suggesting that B1 and B3 ZBLs include at least five and four subgroups, respectively [40]. In addition, improved similarity criteria have been proposed for β-lactamases in general (both zinc-dependent ZBLs and serine-active enzymes), based on *ad hoc* HMM profiles [41]. The results presented here as a Pfam-based SSN and phenetic-cladistic phylogeny comparisons are consistent with those findings, stressing that B1 and B2 enzymes are more related to flavodiiron proteins (FDPs, a group of non-heme iron flavoenzymes) and alkylsulfatases, than to B3 ZBLs. FDPs like *Desulfovibrio gigas* rubredoxin:oxygen oxidoreductase ROO (Figure 1) [42] comprise a widespread family of prokaryotic oxidoreductases, containing an iron-binding MBL domain and an FMN-binding flavodoxin-like domain [43]. ROO is a terminal reductase, which reduces O_2_ to H_2_O without the risk of producing reactive oxygen species. Other structurally characterized FDPs include *Moorella thermoacetica* and *Escherichia coli* nitric oxide reductases, and the *Giardia intestinalis* oxygen-scavenging enzyme. A typical His-to-Glu mutation appears at motif 2 of FDPs, located at the interface between the isoalloxazine and di-iron moieties, which likely contributes to hold the more acidic Fe(III) species. An unusual metal coordination set is found in *Thermotoga maritima* diiron oxygen sensor ODP [44], where the third histidine of motif 2 is replaced by a glutamine at motif 5. Finally, the divergent class-C type-2 FDPs from *Synechocystis* sp. display mutations at motifs 2, 3 and 4 that prevent binding of any metal ions [45].

**Figure 3:**
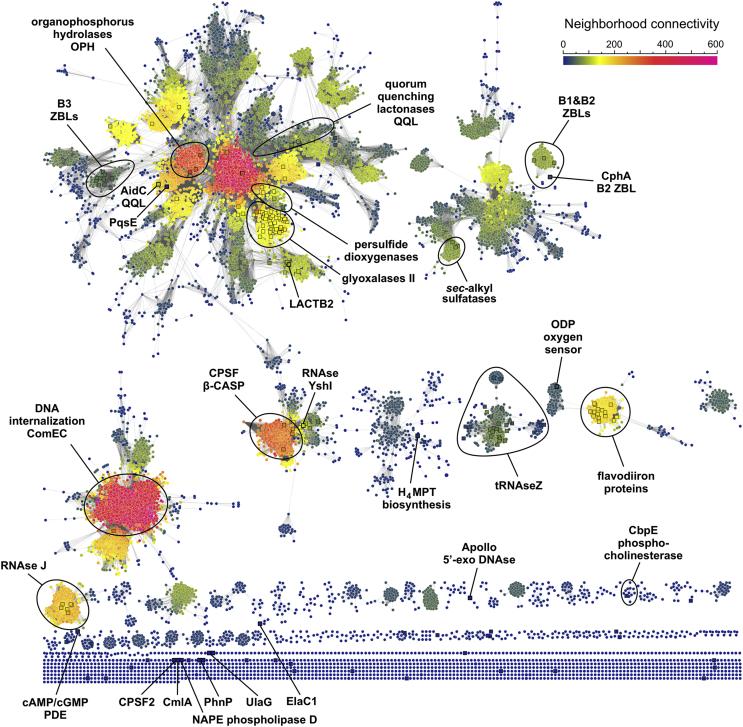
Sequence similarity network (SSN) for representative MBL αββα domains in the Pfam PF00753 database. The network comprises the amino acid sequence of 32,418 MBLs, expressed as 15,292 representative nodes, grouping connected nodes sharing at least 40 % sequence similarity (each representative node size is scaled by the number of proteins included). Edges between pairs of representative nodes indicate a Blast –log(*E-*value) of 20 or better, which corresponds to a sequence identity of at least ~ 30 %. Note that only MBL αββα domains were considered for Blast score calculations. For comparison, proteins and families included in the tanglegram are indicated. Square nodes indicate sequences with SwissProt and/or PDB descriptions (see Supplementary Figure). Note that many structurally and functionally characterized proteins do not cluster with the major components of the SSN but are located in isolated components (*bottom*), since their sequence similarity with proteins in major components is on average lower than 30 %. Nodes are organized with the Cytoscape Prefuse Force Directed Open CL layout, and colored by neighborhood connectivity (*top right*). See Supplementary Spreadsheets S1 and S2, and Supplementary Network for further details.

As shown in Figure 3, alkylsulfatases belong to the same connected component as B1&B2 ZBLs. Type III sulfatases hydrolyze sulfate esters releasing HSO_4_^−^ and the corresponding alcohol. While *Pseudomonas aeruginosa* SdsA1 [46] has preference for primary alcohol sulfates like sodium dodecylsulfate, *Pseudomonas* sp. DSM661 Pisa1 is active on secondary alcohol sulfates, which allowed the discovery that the reaction proceeds with inversion of configuration [47]. Hydrolysis of a secondary alcohol sulfate can proceed through cleavage of C–O or O–S bonds, by nucleophilic attack on the C or S atom, respectively, but only the former can result in inversion of configuration. This is an unprecedented reaction mechanism in the MBL superfamily because the nucleophilic attack occurs on the alcohol carbon by means of an S_N_2 concerted reaction, where HSO_4_^−^ is the leaving group. Thus, MBL *sec*-alkylsulfatases are highly enantioselective enzymes with great potential for application to deracemization processes [48]. In this group, there is also a clade of prokaryotic MBLs of unknown function; the human mitochondrial endoribonuclease LACTB2; and *Pseudomonas* sp. quinolone response protein PqsE. LACTB2 has been shown to use Zn(II) to hydrolyze ssRNA [49]; likely involved in RNA processing specific to mitochondrial function due to its localization and structural homology with bacterial enzymes. PqsE has been shown to bind Fe(II)/Fe(III) *in vitro* and display thiolesterase activity against a CoA-linked intermediate in the biosynthetic pathway of quinolone quorum sensing molecules, although it also contributes to the regulation of bacterial virulence through an unknown mechanism, unrelated to its thiolesterase function [50].

Glyoxalases II (GlxII) and persulfide dioxygenases (PSDO) share a structurally homologous MBL domain, suggestive of common ancestry. This can also be witnessed in the MSA cladogram, where this group forms a separate clade. Human glyoxalase II was the first prototypical MBL to be characterized through X-ray crystallography, disclosing the typical structural features of MBLs. GlxII are thiolesterases that convert *S*-D-lactoylglutathione into D-lactate and glutathione, as part of a ubiquitous methylglyoxal detoxification pathway [51]. The enzyme contains an αββα domain with a consensus HxHxDH motif for binding of two metal ions, reportedly Zn(II) or Mn(II), with an aspartic acid bridge in between. An additional C-terminal domain enables the enzyme to recognize and orient the glutathione moiety for proper hydrolysis, which takes place in the MBL domain metal-binding site. PSDOs are also named ETHE after the human ethylmalonic encephalopathy, a disease that has been linked to mutant PSDO enzymes [52]. Strikingly, while GlxII enzymes harbor a conventional MBL bimetallic center, PSDO enzymes have a single Fe(III) ion at site 1, even though all anticipated metal binding motif residues are conserved. Nevertheless, both enzyme groups catalyze reactions involving glutathione derivatives, *e.g.* 2-hydroxyacyl-glutathione for GlxII and glutathione-persulfide (GSS^–^) for PSDOs, which detoxify sulfide by oxidation to sulfite using molecular oxygen [53]. Some PSDO enzymes like the *Burkholderia phytofirmans* enzyme are fused to rhodanese domains, working instead in sulfur assimilation pathways [54].

The next group comprises at least three phylogenetically distinct structural homologs: quorum-quenching lactonases (QQL), organophosphorus hydrolases (OPH), and human MBLAC1 endonuclease. A number of phenotypes exhibited by bacterial communities are regulated by freely diffusing small molecules signaling cell density. This quorum sensing mechanism is turned off by QQL enzymes like *Bacillus thuringensis* AiiA and *Agrobacterium* sp. AiiB, acting on *N*-acylhomoserine lactones; *Mesorhizobium japonicum* lactonase acting on 4-pyridoxolactone (an intermediate of vitamin B_6_ catabolism); and *Chriseobacterium* sp. AidC lactonase. OPH enzymes like *Pseudomonas* sp. OPHC2 and methylparathion hydrolase MPH are related to QQLs but evolved to hydrolyze phosphoester bonds habitually present in organophosphorus pesticides. Indeed, OPHs may have evolved from QQLs as a resistance mechanism due to the strong selective pressure of these pesticides, resembling how ZBLs evolved to hydrolyze β-lactam antibiotics. Finally, MBLAC1 is a metazoan 3'-end mRNA processing enzyme, acting on stem-loop structures present in histone coding mRNAs [55], constituting the first of many examples of MBL nucleases.

Phosphoesterases comprise the most widespread functional group of the MBL superfamily, hydrolyzing varied phosphoesters like nucleic acids and nucleotides, phosphonates, and phospholipids. Nucleic acid processing enzymes are usually binuclear Zn(II)-dependent hydrolases, such as RNAse J, tRNAse Z, cleavage and polyadenylation specificity factors (CPSF); and DNA repair enzymes like Apollo 5’-exonuclease. These enzymes typically comprise additional domains in a modular fashion that assist the αββα hydrolytic domain at accommodating such large substrates, for instance, the tRNAse Z exosite for tRNA binding [56], β-CASP domains for binding of RNA and DNA [57] (Figure 1), and KH domains for RNA/DNA binding [58]. These modular domains can be either N-terminal, C-terminal, or inserted within the MBL fold. Indeed, the β-CASP domain sequence inserts in the loop holding the conserved His at motif 5, shifting this amino acid about 215 residues towards the C-terminus, making it difficult to find through conventional sequence alignments (*e*.*g*. *T. thermophilus* RNAse J). Analogously, the exosite insertion in tRNAse Z shifts the His at motif 5 about 75 residues to the C-terminus (*e*.*g*. *E. coli* ZipD). The yeast Trz1 tRNAse Z is an interesting example of a protein with two MBL domains where one of them evolved to improve substrate binding while losing the metal-binding and hydrolytic ability [59] (note that only the catalytic domain of Trz1 was considered in the alignment of Figure 2).

Structurally characterized phosphoesterases devoid of nuclease activity include diverse enzymes like *S. pneumoniae* modular phosphorylcholine esterase CbpE; human *N*-acyl phosphatidyl ethanolamine phospholipase D, NAPE-PLD (the only structurally characterized MBL phospholipase), and di-manganese phosphonatase PhnP from *E. coli*, part of the phosphorus scavenging CP-lyase pathway. Note that PhnP are structurally and phylogenetically related to tRNAse Z enzymes, despite their radically different functions. *Streptococcus pneumoniae* phosphoryl-cholinesterase CbpE is localized in the pneumococcal cell envelope [60], and catalyzes the removal the phosphorylcholine from teichoic acids, key components for cell recognition and invasiveness. The divergent *E. coli* manganese-dependent UlaG L-ascorbate-6-P lactonase clusters among phosphoesterases, and has indeed been shown to hydrolyze cyclic nucleotides [61].

Some divergent iron-dependent oxidoreductases cluster at the end of the tanglegram, including *Thermoanaerobacter tengcongensis (C. subterraneus)* Tflp, and *Streptomyces venezuelae* CmlA β-hydroxylase. Tflp contains two Cys residues in the vicinity of the di-iron center, with an Asp-to-Cys mutation at motif 4 (seen so far only in modern B1&B2 zinc-β-lactamases), plus a unique Cys residue following the His residue at motif 5. Complementary spectroscopic assays indicate that Tflp holds an [Fe–S] center under reducing conditions, and structure PDB 2p4z corresponds to an oxidized inactive form. On the other hand, CmlA is a rare β-hydroxylase clustering among phosphoesterases, which hydroxylates L-*p*-aminophenylalanine, a biosynthetic precursor of chloramphenicol.

### 3.3 SSN analysis suggests that numerous MBL families remain to be characterized

**Figure 4:**
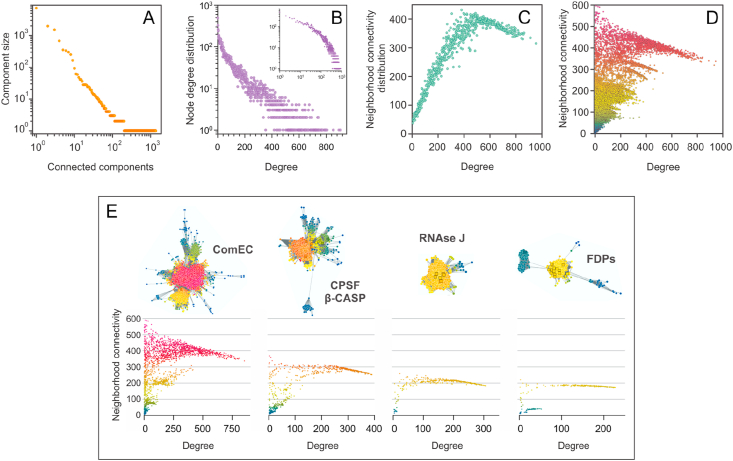
Topological parameters of the MBL superfamily SSN. (A) Connected components (CC) are sets of nodes connected by paths of edges. Although a full SSN comprises a single CC, setting an alignment score cutoff leads to a disconnected network aiming to isolate individual protein families, and thereby a set of CCs. The distribution of CC sizes approximately follows a power law, *i.e.* a straight line with negative slope in log-log scale. (B) Many natural networks follow a power law distribution of node degrees. However, the SSN node degree distribution is convex up and skewed toward highly connected nodes or nodes with relatively large neighborhoods (Box 1). (C) If all NC values are averaged for each degree value, the NC distribution is obtained. A maximum neighborhood connectivity of ~ 400 is observed for *k* ~ 500, which means that, on average, neighborhoods larger or smaller than ~ 500 neighbors are less interconnected. (D) Plotting all NC values for each node degree results in a scatter plot with “spikes” for highly interconnected clusters, *i.e.* highly similar groups of proteins (compare with Figure 3, the same coloring was used here). (E) Plots of NC *vs.* node degree for individual CCs provide a clearer picture of how NC values show an almost inverse linear relationship with connectivity, skewed to larger connectivity values.

An SSN was here calculated for the MBL superfamily using the EFI-EST webserver [33], as described in the Methods section; results are shown in Figure 3 (see Supplementary Spreadsheet S1 and Supplementary Network for full network data). SSNs are graphs with nodes representing protein sequences and edges connecting them, indicating a pairwise sequence similarity at a specified cutoff value. The metric for node similarity calculation at EFI-EST is the Blast *E*-value, which was set to –log(*E*-value) = 20. Unless otherwise stated, nodes are specifically representative nodes, which group several UniProt entries with a 40 % or higher sequence similarity, so that the SSN has fewer edges and is simpler to display graphically. By inspecting the distribution of functionally characterized proteins throughout the SSN it is evident that many MBL families remain to be characterized. In fact, one of the largest clusters in the network comprises proteins involved in DNA internalization and natural competence such as ComEC, for which no structural information is yet available and only one SwissProt entry (*Bacillus subtilis* P39695) is described. The size of connected components (CC) in the SSN follows a power law distribution, with a few clusters encompassing most nodes, and a long tail of many CCs with one or two nodes (Figure 4A). The largest CC (7259 nodes) includes glyoxalases II, PSDOs, OPHs, QQLs and B3 ZBLs; the second (1962 nodes) includes B1&B2 ZBLs and *sec*-alkyl sulfatases; and the third (1503 nodes) DNA internalization/ComEC proteins; whereas CPSF/β-CASP, tRNAse Z, RNAse J, and FDPs cluster into separate CCs of 673, 350, 333 and 297 nodes, respectively. The remaining 2915 nodes (19 %) include relatively few known MBLs sparsely scattered over 1353 smaller CCs. Analogously, the node degree shows a sharply decaying distribution, skewed towards lowly connected nodes (Figure 4B). This is probably true for all SSNs for a given alignment score cutoff, since new nodes (proteins) likely become part of existing connected components (families) instead of giving rise to new ones. Nevertheless, the curve is convex up in log-log scale (*inset*), *i.e.* it is not a power law distribution. Only 148 nodes have SwissProt descriptions and 91 nodes have at least one PDB experimentally determined structure (41 nodes have both). As depicted in Figure 3, the majority of nodes with SwissProt and PDB entries describe glyoxalases II, ribonucleases, FDPs, and ZBLs, accounting for 198 out of 15,292 nodes (1.3 %). In other words, 98.7 % of the SSN nodes need experimentally obtained functional and/or structural information so that an accurate annotation can be specified. Given the fast pace at which sequence databases grow, misannotation of macromolecular sequences is an increasingly cumbersome problem [62, 63, 64], and relying on entry annotations to define protein families is not a judicious approach.

### 3.4 Neighborhood connectivity distribution correlates with protein family clustering

The neighborhood connectivity (NC) statistic was introduced in 2002 by Maslov & Sneppen to describe how sets of highly connected regulatory genes control the expression of lowly connected genes [21] (Box 1). In SSNs, highly interconnected clusters share sequence and, presumably, functional similarity. Thus, members of protein families should have similar connectivities, and coloring nodes by NC provides an intuitive way of visually spotting protein families within CCs. Highly interconnected clusters indicate conserved, highly similar sequences; whereas lowly connected nodes point to rare sequences, proteins underrepresented in the SSN, or simply noise (*e.g*. truncated or incomplete sequences). For a given set of protein sequences, the SSN topology often matches the corresponding phylogenetic tree topology [12]; however, such agreement depends critically on the metrics used for network, MSA, and tree calculation [65]. This is particularly important when comparing divergent sequences sharing few conserved motifs, like the MBL superfamily. For instance, while functional families cluster into distinct clades in the tanglegram, the SSN largest connected component includes most lactonases, glyoxalases II, PSDOs, and B3 ZBLs; and separate clusters are observed for tRNAse Z, RNAse J, and CPSF phosphoesterases (Figure 3). Besides, while B1&B2 ZBLs cluster with alkylsulfatases in the SSN, the tanglegram shows that FDPs are their closest structural homologs. These apparent discrepancies likely reflect the different calculation metrics, *i.e.* Blast *E*-value for the SSN as opposed to structural homology for the tanglegram. The NC distribution reaches a maximum of ~ 400 for nodes with ~ 500 neighbors (Figure 4C), decaying almost linearly for higher connectivities. Apparently, once clusters reach a maximal connectivity or edges per node, they grow upon addition of new nodes but fewer connections are introduced. This reciprocal linear relationship observed for the full network seems to hold true also for individual clusters: plotting individual NC values reveals linear segments for each cluster, provided that enough nodes are present (Figures 4D&E). These features likely reflect the network topology arising from using the Blast *E*-value as a metric for sequence comparison, which ultimately defines the lengths of edges connecting nodes within CCs. A detailed description of these curves requires further research on SSN properties, which will shed light on the dynamics of protein network growth and degree distributions.

**Box 1:**
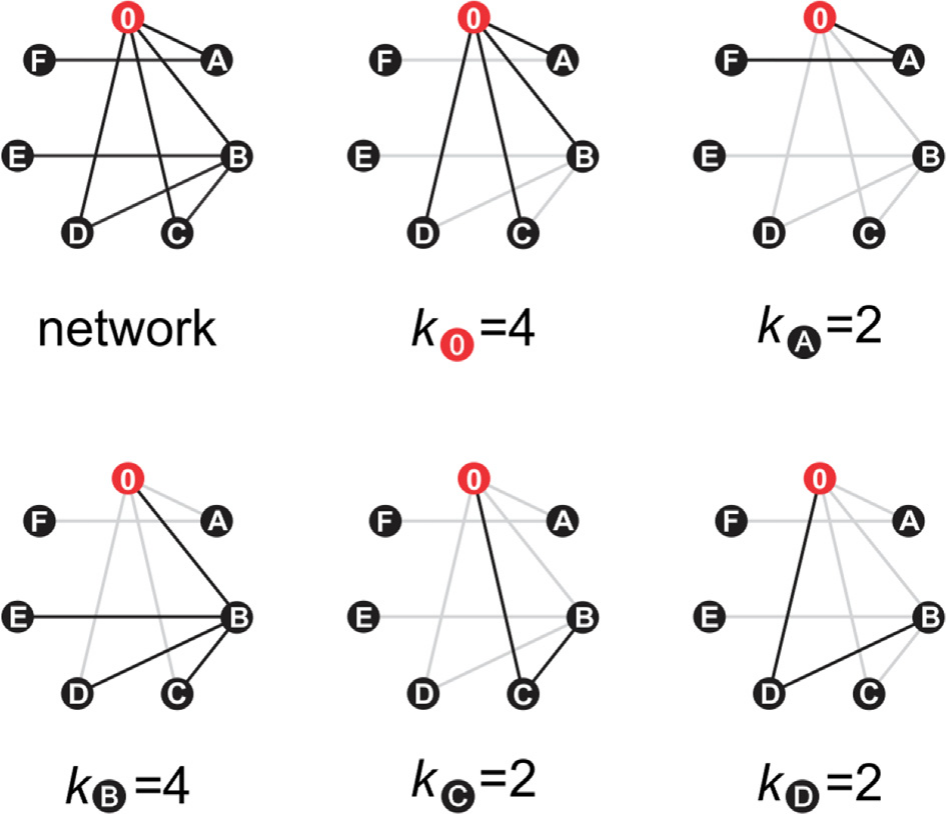
Neighborhood connectivity. Unlike many so-called “biological networks” such as protein-protein interaction networks or metabolic networks, SSNs are undirected and do not display self-edges. Then, the *neighborhood* of a node *n* is the set of nodes sharing an edge with *n*; and its *connectivity*, *k*_n_, is the size of its neighborhood, *i.e.* the number of neighbors of *n*. The *degree* of node *n* is the number of edges reaching *n*, which is equivalent to *k*_n_ for SSNs. Then, the *neighborhood connectivity* (NC) of *n*, is defined as the average connectivity of its neighborhood, NC_n_ = Σ(*k*_i_)/*k*_n_ [21, 34]. For example, for a given node 0 (*red*) in the network {0, A, B, C, D, E, F} (*top left*), the neighborhood of 0 is {A, B, C, D} of size *k*_0_ = 4, and the connectivities of each of its neighbors are *k*_A_ = 2, *k*_B_ = 4, *k*_C_ = 2, and *k*_D_ = 2. Then, the neighborhood connectivity of 0 is NC_0_ = (*k*_A_ + *k*_B_ + *k*_C_ + *k*_D_)/*k*_0_ = 2.5. Note that even though nodes E and F are not neighbors of 0, they still influence its NC value by increasing *k*_A_ and *k*_B_. Since members of a protein family are expected to cluster together sharing edges with each other, their neighborhood connectivities will exhibit comparable values. This can be readily appreciated in Figure 3 by coloring nodes according to their NC values. If *N* nodes in an isolated cluster are connected all-to-all, for each node *k* = *N* − 1 (neighbors or edges), all nodes will have a neighbor connectivity NC = *N* – 1. For example, if the network {0, A, B, C, D, E, F} had edges connecting all-to-all its *N* = 7 nodes, each node would have *k* = NC = 6 neighbors (~ *N* for large clusters). In other words, for highly interconnected clusters, the neighbor connectivity approaches to its maximum value, which is roughly the size of the cluster (*cf*. Figure 4 C).

## 4 Concluding remarks

Herein, structural homology and SSN analysis are used to assist the phylogenetic reconstruction of the MBL superfamily, harnessing the protein three-dimensional arrangement of secondary structure elements as a metric for common ancestry inference. The introduced tanglegram graph disclosed structure and sequence similarity relationships between seemingly unrelated enzymes, which is suggestive of a mutual evolutionary history. Tanglegrams comprise a practical framework for protein structure-function analysis, applicable to study other protein superfamilies as well. Analogously, NC network coloring provides an intuitive picture of the distribution of protein families within the superfamily, suggesting that numerous MBL families remain to be characterized. Indeed, manually annotated entries for proteins with available experimental evidence account for only 1.3 % of the superfamily, underscoring an unfortunately frequent bias of research towards relatively few families. Automated annotation algorithms would benefit from further research on protein SSNs; establishing their topological features will give rise to improved metrics for protein function estimation.

## Declarations

### Author contribution statement

Javier M González: Conceived and designed the experiments; Performed the experiments; Analyzed and interpreted the data; Contributed reagents, materials, analysis tools or data; Wrote the paper.

### Funding statement

This work was supported by Agency for Science and Technology Promotion (ANPCyT), grant PICT 2017-4590, Argentina.

### Data availability statement

Data included in article/supplementary material/referenced in article.

### Declaration of interests statement

The authors declare no conflict of interest.

### Additional information

No additional information is available for this paper.

## Appendix A Supplementary data

Supplementary Figure.pdf Supplementary Figure.pdf

Supplementary Network.cys MBL superfamily SSN (as shown in Figure 3), displayed with Cytoscape 3.8.2 (available at https://cytoscape.org/). Supplementary Network.cys

Supplementary Spreadsheet S1.xlsx Full entry data of the 15294 representative nodes included in the MBL superfamily SSN (as shown in Figure 3). Supplementary Spreadsheet S1.xlsx

Supplementary Spreadsheet S2.xlsx Detailed list of Protein Data Bank (PDB) entries present in the MBL superfamily **SSN** (as shown in Figure 3). The 292 PDB entries can be mapped to 100 UniProt entries. Supplementary Spreadsheet S2.xlsx

## References

[1] Carfi A., Pares S., Duée E., Galleni M., Duez C., Frère J.M., Dideberg O. (1995). The 3-D structure of a zinc metallo-beta-lactamase from Bacillus cereus reveals a new type of protein fold. EMBO J..

[2] Concha N.O., Rasmussen B.A., Bush K., Herzberg O. (1996). Crystal structure of the wide-spectrum binuclear zinc beta-lactamase from Bacteroides fragilis. Structure.

[3] Fabiane S.M., Sohi M.K., Wan T., Payne D.J., Bateson J.H., Mitchell T., Sutton B.J. (1998). Crystal structure of the zinc-dependent beta-lactamase from Bacillus cereus at 1.9 A resolution: binuclear active site with features of a mononuclear enzyme. Biochemistry.

[4] Carfi A., Duée E., Galleni M., Frère J.M., Dideberg O. (1998). 1.85 A resolution structure of the zinc (II) beta-lactamase from Bacillus cereus. Acta Crystallogr D Biol Crystallogr.

[5] Orellano E.G., Girardini J.E., Cricco J.A., Ceccarelli E.A., Vila A.J. (1998). Spectroscopic characterization of a binuclear metal site in Bacillus cereus beta-lactamase II.. Biochemistry.

[6] Llarrull L.I., Tioni M.F., Kowalski J., Bennett B., Vila A.J. (2007). Evidence for a dinuclear active site in the metallo-beta-lactamase BcII with substoichiometric Co(II). A new model for metal uptake. J. Biol. Chem..

[7] González J.M., Meini M.-R., Tomatis P.E., Martín F.J.M., Cricco J.A., Vila A.J. (2012). Metallo-β-lactamases withstand low Zn(II) conditions by tuning metal-ligand interactions. Nat. Chem. Biol..

[8] Aravind L. (1999). An evolutionary classification of the metallo-beta-lactamase fold proteins. Silico Biol..

[9] Altschul S.F., Madden T.L., Schäffer A.A., Zhang J., Zhang Z., Miller W., Lipman D.J. (1997). Gapped BLAST and PSI-BLAST: a new generation of protein database search programs. Nucleic Acids Res..

[10] Daiyasu H., Osaka K., Ishino Y., Toh H. (2001). Expansion of the zinc metallo-hydrolase family of the beta-lactamase fold. FEBS Lett..

[11] Finn R.D., Attwood T.K., Babbitt P.C., Bateman A., Bork P., Bridge A.J., Chang H.-Y., Dosztányi Z., El-Gebali S., Fraser M., Gough J., Haft D., Holliday G.L., Huang H., Huang X., Letunic I., Lopez R., Lu S., Marchler-Bauer A., Mi H., Mistry J., Natale D.A., Necci M., Nuka G., Orengo C.A., Park Y., Pesseat S., Piovesan D., Potter S.C., Rawlings N.D., Redaschi N., Richardson L., Rivoire C., Sangrador-Vegas A., Sigrist C., Sillitoe I., Smithers B., Squizzato S., Sutton G., Thanki N., Thomas P.D., Tosatto S.C.E., Wu C.H., Xenarios I., Yeh L.-S., Young S.-Y., Mitchell A.L. (2017). InterPro in 2017-beyond protein family and domain annotations. Nucleic Acids Res..

[12] Atkinson H.J., Morris J.H., Ferrin T.E., Babbitt P.C. (2009). Using sequence similarity networks for visualization of relationships across diverse protein superfamilies. PloS One.

[13] Atkinson H.J., Babbitt P.C. (2009). An atlas of the thioredoxin fold class reveals the complexity of function-enabling adaptations. PLoS Comput. Biol..

[14] Baier F., Tokuriki N. (2014). Connectivity between catalytic landscapes of the metallo-β-lactamase superfamily. J. Mol. Biol..

[15] Davidson R., Baas B.-J., Akiva E., Holliday G.L., Polacco B.J., LeVieux J.A., Pullara C.R., Zhang Y.J., Whitman C.P., Babbitt P.C. (2018). A global view of structure-function relationships in the tautomerase superfamily. J. Biol. Chem..

[16] Copp J.N., Anderson D.W., Akiva E., Babbitt P.C., Tokuriki N. (2019). Exploring the sequence, function, and evolutionary space of protein superfamilies using sequence similarity networks and phylogenetic reconstructions. Methods Enzymol..

[17] Malik A., Kim S.B. (2019). A comprehensive in silico analysis of sortase superfamily. J. Microbiol..

[18] Shi Q., Wang H., Liu J., Li S., Guo J., Li H., Jia X., Huo H., Zheng Z., You S., Qin B. (2020). Old yellow enzymes: structures and structure-guided engineering for stereocomplementary bioreduction. Appl. Microbiol. Biotechnol..

[19] Tararina M.A., Allen K.N. (2020). Bioinformatic analysis of the flavin-dependent amine oxidase superfamily: adaptations for substrate specificity and catalytic diversity. J. Mol. Biol..

[20] Easley D., Kleinberg J. (2010). Networks, crowds, and markets: reasoning about a highly connected world.

[21] Maslov S. (2002). Specificity and stability in topology of protein networks. Science (80- ).

[22] Holm L., Laakso L.M. (2016). Dali server update. Nucleic Acids Res..

[23] Holm L. (2020). Using Dali for protein structure comparison.

[24] The Uniprot Consortium & Bateman A (2019). UniProt: a worldwide hub of protein knowledge. Nucleic Acids Res..

[25] Pei J., Tang M., Grishin N.V. (2008). PROMALS3D web server for accurate multiple protein sequence and structure alignments. Nucleic Acids Res..

[26] Waterhouse A.M., Procter J.B., Martin D.M.A., Clamp M., Barton G.J. (2009). Jalview Version 2–a multiple sequence alignment editor and analysis workbench. Bioinformatics.

[27] Stamatakis A. (2014). RAxML version 8: a tool for phylogenetic analysis and post-analysis of large phylogenies. Bioinformatics.

[28] Miller M.A., Pfeiffer W., Schwartz T. (2010). Creating the CIPRES Science Gateway for inference of large phylogenetic trees. 2010 Gateway Computing Environments Workshop (GCE).

[29] Whelan S., Goldman N. (2001). A general empirical model of protein evolution derived from multiple protein families using a maximum-likelihood approach. Mol. Biol. Evol..

[30] Scornavacca C., Zickmann F., Huson D.H. (2011). Tanglegrams for rooted phylogenetic trees and networks. Bioinformatics.

[31] Huson D.H., Scornavacca C. (2012). Dendroscope 3: an interactive tool for rooted phylogenetic trees and networks. Syst. Biol..

[32] Huang Y., Niu B., Gao Y., Fu L., Li W. (2010). CD-HIT Suite: a web server for clustering and comparing biological sequences. Bioinformatics.

[33] Gerlt J.A., Bouvier J.T., Davidson D.B., Imker H.J., Sadkhin B., Slater D.R., Whalen K.L. (2015). Enzyme Function Initiative-Enzyme Similarity Tool (EFI-EST): a web tool for generating protein sequence similarity networks. Biochim. Biophys. Acta Protein Proteonomics.

[34] Doncheva N.T., Assenov Y., Domingues F.S., Albrecht M. (2012). Topological analysis and interactive visualization of biological networks and protein structures. Nat. Protoc..

[35] Shannon P. (2003). Cytoscape: a software environment for integrated models of biomolecular interaction networks. Genome Res..

[36] Garau G., Di Guilmi A.M., Hall B.G. (2005). Structure-based phylogeny of the metallo-β-lactamases. Antimicrob. Agents Chemother..

[37] Galleni M., Lamotte-Brasseur J., Rossolini G.M., Spencer J., Dideberg O., Frère J.-M. (2001). standard numbering scheme for class B β-lactamases. Antimicrob. Agents Chemother..

[38] Hall B.G., Salipante S.J., Barlow M. (2003). The metallo-beta-lactamases fall into two distinct phylogenetic groups. J. Mol. Evol..

[39] Garau G., García-Sáez I., Bebrone C., Anne C., Mercuri P., Galleni M., Frère J.-M., Dideberg O. (2004). Update of the standard numbering scheme for class B β-lactamases. Antimicrob. Agents Chemother..

[40] Berglund F., Johnning A., Larsson D.G.J., Kristiansson E. (2020). An updated phylogeny of the metallo-β-lactamases. J. Antimicrob. Chemother..

[41] Silveira M.C., Azevedo da Silva R., Faria da Mota F., Catanho M., Jardim R., Guimarães A.C.R., de Miranda A.B. (2018). Systematic identification and classification of β-lactamases based on sequence similarity criteria: β-lactamase annotation. Evol. Bioinf. Online.

[42] Frazão C., Silva G., Gomes C.M., Matias P., Coelho R., Sieker L., Macedo S., Liu M.Y., Oliveira S., Teixeira M., Xavier A.V., Rodrigues-Pousada C., Carrondo M.A., Le Gall J. (2000). Structure of a dioxygen reduction enzyme from Desulfovibrio gigas. Nat. Struct. Biol..

[43] Vicente J.B., Carrondo M.A., Teixeira M., Frazão C. (2008). Structural studies on flavodiiron proteins. Methods Enzymol..

[44] Muok A.R., Deng Y., Gumerov V.M., Chong J.E., DeRosa J.R., Kurniyati K., Coleman R.E., Lancaster K.M., Li C., Zhulin I.B., Crane B.R. (2019). A di-iron protein recruited as an Fe[II] and oxygen sensor for bacterial chemotaxis functions by stabilizing an iron-peroxy species. Proc. Natl. Acad. Sci. Unit. States Am..

[45] Borges P.T., Romão C.V., Saraiva L.M., Gonçalves V.L., Carrondo M.A., Teixeira M., Frazão C. (2019). Analysis of a new flavodiiron core structural arrangement in Flv1-ΔFlR protein from Synechocystis sp. PCC6803. J. Struct. Biol..

[46] Hagelueken G., Adams T.M., Wiehlmann L., Widow U., Kolmar H., Tummler B., Heinz D.W., Schubert W.-D. (2006). The crystal structure of SdsA1, an alkylsulfatase from Pseudomonas aeruginosa, defines a third class of sulfatases. Proc. Natl. Acad. Sci. Unit. States Am..

[47] Knaus T., Schober M., Kepplinger B., Faccinelli M., Pitzer J., Faber K., Macheroux P., Wagner U. (2012). Structure and mechanism of an inverting alkylsulfatase from Pseudomonas sp. DSM6611 specific for secondary alkyl sulfates. FEBS J..

[48] Schober M., Gadler P., Knaus T., Kayer H., Birner-Grünberger R., Gülly C., Macheroux P., Wagner U., Faber K. (2011). A stereoselective inverting sec -alkylsulfatase for the deracemization of sec -alcohols. Org. Lett..

[49] Levy S., Allerston C.K., Liveanu V., Habib M.R., Gileadi O., Schuster G. (2016). Identification of LACTB2, a metallo-β-lactamase protein, as a human mitochondrial endoribonuclease. Nucleic Acids Res..

[50] Zender M., Witzgall F., Drees S.L., Weidel E., Maurer C.K., Fetzner S., Blankenfeldt W., Empting M., Hartmann R.W. (2016). Dissecting the multiple roles of PqsE in Pseudomonas aeruginosa virulence by discovery of small tool compounds. ACS Chem. Biol..

[51] Cameron A.D., Ridderström M., Olin B., Mannervik B. (1999). Crystal structure of human glyoxalase II and its complex with a glutathione thiolester substrate analogue. Structure.

[52] Tiranti V., Viscomi C., Hildebrandt T., Di Meo I., Mineri R., Tiveron C., Levitt M.D., Prelle A., Fagiolari G., Rimoldi M., Zeviani M. (2009). Loss of ETHE1, a mitochondrial dioxygenase, causes fatal sulfide toxicity in ethylmalonic encephalopathy. Nat. Med..

[53] Zhang L., Liu X., Qin Z., Liu J., Zhang Z. (2016). Expression characteristics of sulfur dioxygenase and its function adaption to sulfide in echiuran worm Urechis unicinctus. Gene.

[54] Motl N., Skiba M.A., Kabil O., Smith J.L., Banerjee R. (2017). Structural and biochemical analyses indicate that a bacterial persulfide dioxygenase–rhodanese fusion protein functions in sulfur assimilation. J. Biol. Chem..

[55] Pettinati I., Grzechnik P., Ribeiro de Almeida C., Brem J., McDonough M.A., Dhir S., Proudfoot N.J., Schofield C.J. (2018). Biosynthesis of histone messenger RNA employs a specific 3’ end endonuclease. Elife 7.

[56] Schilling O., Späth B., Kostelecky B., Marchfelder A., Meyer-Klaucke W., Vogel A. (2005). Exosite modules guide substrate recognition in the ZiPD/ElaC protein family. J. Biol. Chem..

[57] Callebaut I. (2002). Metallo-beta-lactamase fold within nucleic acids processing enzymes: the beta-CASP family. Nucleic Acids Res..

[58] Grishin N.V. (2001). KH domain: one motif, two folds. Nucleic Acids Res..

[59] Ma M., Li de la Sierra-Gallay I., Lazar N., Pellegrini O., Durand D., Marchfelder A., Condon C., van Tilbeurgh H. (2017). The crystal structure of Trz1, the long form RNase Z from yeast. Nucleic Acids Res..

[60] Hermoso J.A., Lagartera L., González A., Stelter M., García P., Martínez-Ripoll M., García J.L., Menéndez M. (2005). Insights into pneumococcal pathogenesis from the crystal structure of the modular teichoic acid phosphorylcholine esterase Pce. Nat. Struct. Mol. Biol..

[61] Garces F., Fernández F.J., Montellà C., Penya-Soler E., Prohens R., Aguilar J., Baldomà L., Coll M., Badia J., Vega M.C. (2010). Molecular architecture of the Mn2+-dependent lactonase UlaG reveals an RNase-like metallo-β-lactamase fold and a novel quaternary structure. J. Mol. Biol..

[62] Schnoes A.M., Brown S.D., Dodevski I., Babbitt P.C. (2009). Annotation error in public databases: misannotation of molecular function in enzyme superfamilies. PLoS Comput. Biol..

[63] Liberal R., Pinney J.W. (2013). Simple topological properties predict functional misannotations in a metabolic network. Bioinformatics.

[64] Nobre T., Campos M.D., Lucic-Mercy E., Arnholdt-Schmitt B. (2016). Misannotation awareness: a tale of two gene-groups. Front. Plant Sci..

[65] Leuthaeuser J.B., Knutson S.T., Kumar K., Babbitt P.C., Fetrow J.S. (2015). Comparison of topological clustering within protein networks using edge metrics that evaluate full sequence, full structure, and active site microenvironment similarity. Protein Sci..

