# Supplementary material for: Visualizing the superfamily of metallo-β-lactamases through sequence similarity network neighborhood connectivity analysis
Source: Heliyon. 2021 Jan 2;7(1):e05867. doi: 10.1016/j.heliyon.2020.e05867 (PMC7785958; doi:10.1016/j.heliyon.2020.e05867)

Supplementary Figure

Sequence similarity network (as shown in Figure 3) for representative MBL αββα domains in the Pfam database PF00753, highlighting representative nodes comprising entries from SwissProt and PDB databases.

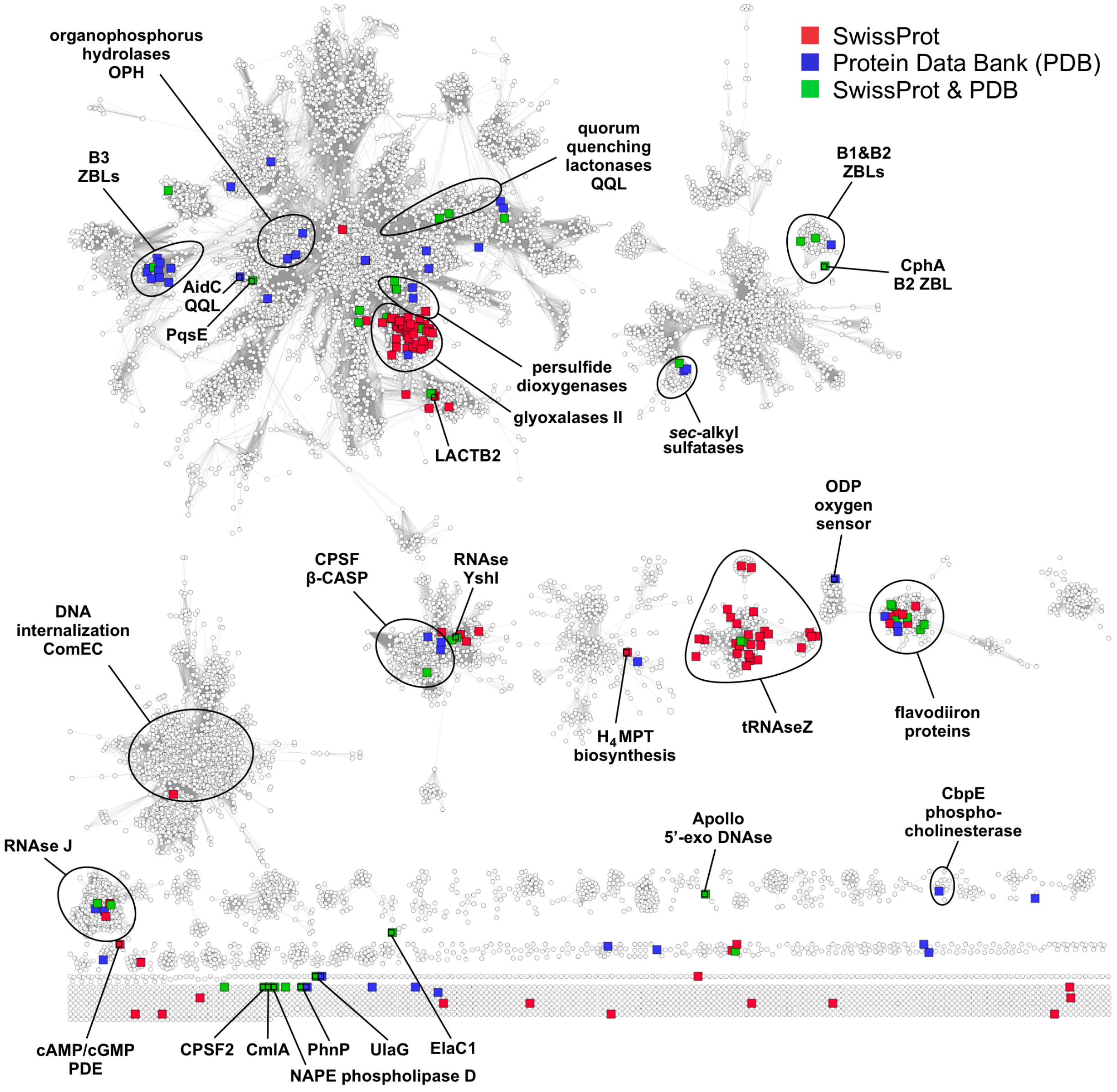

Supplement: Supplementary Figure.pdf [file mmc1.pdf]
